# A Preclinical Pilot Study on the Effects of Thermal Ablation on Lamb Growth Plates

**DOI:** 10.3390/children9060878

**Published:** 2022-06-12

**Authors:** Katharina Jäckle, Sebastian Lippross, Theresa Elisabeth Michel, Johannes T. Kowallick, Christian Dullin, Katja A. Lüders, Heiko M. Lorenz, Konstantinos Tsaknakis, Anna K. Hell

**Affiliations:** 1 Pediatric Orthopaedics, Department for Trauma Surgery, Orthopaedics and Plastic Surgery, University Medical Center Göttingen, Robert-Koch Str. 40, 37075 Göttingen, Germany; katharina.jaeckle@med.uni-goettingen.de (K.J.); t.michel01@stud.uni-goettingen.de (T.E.M.); katja.lueders@med.uni-goettingen.de (K.A.L.); heiko.lorenz@med.uni-goettingen.de (H.M.L.); konstantinos.tsaknakis@med.uni-goettingen.de (K.T.); 2 Department for Trauma Surgery, Orthopaedics and Plastic Surgery, University Medical Center Göttingen, Robert-Koch Str. 40, 37075 Göttingen, Germany; 3 Departement of Orthopaedics and Trauma Surgery, University Medical Center Schleswig-Holstein, Arnold-Heller-Str. 3, 24105 Kiel, Germany; sebastian.lippross@uksh.de; 4 Institute for Diagnostic and Interventional Radiology, University Medical Center Göttingen, Robert-Koch Str. 40, 37075 Göttingen, Germany; johannes.kowallick@med.uni-goettingen.de (J.T.K.); christian.dullin@med.uni-goettingen.de (C.D.); 5 Institute for Diagnostic und Interventional Radiology, University Hospital Heidelberg, Im Neuenheimer Feld 400, 69120 Heidelberg, Germany

**Keywords:** thermal ablation, accuracy, histology, linseed sheep, growth plate, growth modulation

## Abstract

(1) Background: Thermal ablation has been demonstrated to affect the bone growth of osteoid osteoma in adolescents. Growth modulation due to thermal heat in children is conceivable, but has not yet been established. We used lamb extremities as a preclinical model to examine the effect of thermal ablation on growth plates in order to evaluate its potential for axial or longitudinal growth modulation in pediatric patients. (2) Methods: Thermal ablation was performed by electrocautery on eight different growth plates of the legs and distal radii of a stillborn lamb. After treatment, target hits and the physical extent of the growth plate lesions were monitored using micro-computed tomography (micro-CT) and histology. (3) Results: Lesions and their physical extent could be quantified in 75% of the treated extremities. The histological analysis revealed that the disruption of tissue was confined to a small area and the applied heat did not cause the entire growth plate to be disrupted or obviously damaged. (4) Conclusions: Thermal ablation by electrocautery is minimally invasive and can be used for targeted disruption of small areas in growth plates in the animal model. The results suggest that thermal ablation can be developed into a suitable method to influence epiphyseal growth in children.

## 1. Introduction

In children, longitudinal growth of the long tubular bones is regulated by the epiphyseal growth plates. Today, pediatric orthopedic surgeons focus on growth modulation to adjust axis deviations and length discrepancies. Most lower limb growth deformities are idiopathic, congenital, post-inflammatory, or syndromic, yet traumatic damage of the epiphyses may also lead to post-traumatic growth disturbance [1]. Damage to the growth plates can result in both growth acceleration or growth retardation with length discrepancies and axial deviations. Current treatment options focus on the distraction or inhibition of small areas within the growth plate of the still-growing bones in order to achieve local growth modulation, with the focus of this therapy in children being to correct appropriate deformities on the lower extremities. These therapies include percutaneous techniques using screws or drilling to locally inhibit bone growth so as to ideally restore proper limb growth [2]. Using growth inhibition, a temporary or permanent bridging or destruction of the epiphyseal growth plate either on one (hemiepiphyseodesis) or on both sides (epiphyseodesis) can be performed to influence axial and/or longitudinal growth. Planning of the remaining growth is done, for example, with PaleyGrowth (version 3.0), a software that scores body height, limb length, and limb length discrepancy [3], but other methods can be applied as well. Temporary growth guidance is generally provided by implants (e.g., Blount clamp, eight-Plates^®^ (for example company Orthofix), etc.), which can be removed once the correction is achieved. In older children, definite epiphysiodesis as proposed by Phemister [4] and Canale [5] can be performed to stop growth in the desired area. Both methods are serious surgical procedures that involve either removal of already existing bone material or a thorough curettage of the entire growth plate, which must be performed under radiographic guidance [4,5].

The common goal of these procedures is to maintain both extremities at the same growth level or to correct deformities. In permanent bone growth inhibition, the entire growth plate is removed by reaming the joint. This treatment can lead to the formation of hematomas and joint effusions and even fractures. Other complications include superficial wound infections, postoperative exostoses, and paralysis of the peroneal nerve, and the desired complete growth arrest is not achieved. Reversible epiphysiodesis involves implants, and hardware problems such as kinking, migration, or fracture of the staples occur, especially in younger patients. In addition, the metal must be removed with a second surgery once the desired result is achieved. In addition, the implants can lead to angular misalignments, which can be severe and therefore require corrective osteotomies.

Currently, percutaneous epiphysiodesis is widely used for correcting limb inequality. This corrective treatment offers good results, a low complication rate, and aesthetic scars. However, correct compensation of a length discrepancy or axis deviation is only possible if the epiphysiodesis is performed at the right time, but the accuracy of the available bone growth prediction methods is still quite limited. Therefore, reversible methods are currently preferred in the treatment of angular deformities where prediction is most difficult. They can be used safely in very young children and allow early intervention in case of severe deformities.

A currently used minimally invasive treatment method of osteoid osteoma, which is a benign but very painful small bone tumor, involves thermal ablation under radiographic or computed tomography-controlled application [6]. With this treatment, bone tissue is destroyed through the application of targeted heat using electrocautery. It is conceivable that this procedure could also be used to specifically affect the epiphyseal plate of the growing skeleton with minimal local morbidity. In fact, previous studies in rabbits and pigs have shown that radio-frequency ablation affects bone growth differentiation [7], i.e., growth inhibition was achieved by radio-frequency coagulation of the physis [8]. Encouraged by these results, we performed a pilot study to explore the potential of thermal ablation at the growth plates using the extremities of a stillborn lamb as a preclinical model. We chose lamb extremities because their bones are well comparable to those of infants and they have previously been useful for studying the human musculoskeletal system. Here, we show that minimally invasive thermal ablation under radiological control can be used to generate well-targeted small lesions in the growth plates of the extremities, providing a first promising step towards developing a novel method for targeted modulation to adjust axis deviations and length discrepancies of growing bones in young children.

## 2. Materials and Methods

### 2.1. Preparation of the Cadaver for the Hemiepiphyseodesis

**Figure 1:**
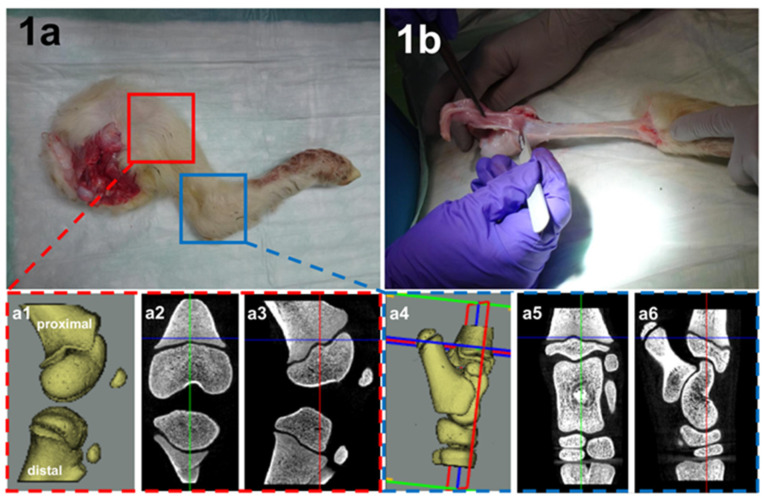
Hind leg separated from the stillborn female linseed sheep (**a**). Anatomical preparation through the muscles up to the appearance of the bone (**b**). The small red box marks the knee joint. The magnification in the dashed box shows a sagittal 3D overview in micro-CT imaging (**a1**), (**a2**) coronal sectioning, and (**a3**) sagittal sectioning through the knee joint. The small blue box marks the ankle. The magnification in the dashed box shows a sagittal 3D overview in the micro-CT imaging (**a4**), (**a5**) coronal sectioning, and (**a6**) sagittal sectioning through the ankle.

The extremities were carefully separated from the stillborn female sheep (animal breed domestic sheep). After shaving, careful layer-by-layer preparation through the muscles up to the appearance of the bone was performed (see Figure 1).

### 2.2. Thermal Ablation Using Electrocautery for Hemiepiphyseodesis

On the radiographs, the following growth plates were identified in all four extremities: (a) distal radius, (b) proximal and (c) distal tibia, and (d) distal femur. The electrode was placed into all four different growth plates per limb under standardized anterior-posterior and lateral radiological control. The very tip of the electrode (0.7 mm; ERBE spatula, 21191-159, 2.3 × 19 mm length, ERBE Elektromedizin GmbH; Tübingen, Germany) was inserted to a depth of 0.5 cm at all eight different growth plates. The electrode heats not only at the tip, but through the whole intracorporal part. Hemiepiphyseodesis, i.e., destruction of the epiphyseal plate on one side, was performed laterally on the corresponding locations of the growth plates. For this purpose, two obliterations (60 s) were carried out with a distance of approx. 1–1.5 cm and with a 80 Watt (approx. 90–95 °C) setting (ERBE ICC 350; ERBE Elektromedizin GmbH; Tübingen, Germany). To determine the location of the ablation in relation to the growth plate and to quantify its spatial extent as a measure of ablation severity, micro-CT was used (see Section 2.3 and Section 2.4 below). As the controls, lesions were placed by tip insertion only without heat application. In each location, two lesions were set with the electrocautery tip per thermal ablation trial, which resulted in a total of 16 lesions in total (see Table 1).

### 2.3. Micro-CT Measurements

**Figure 2:**
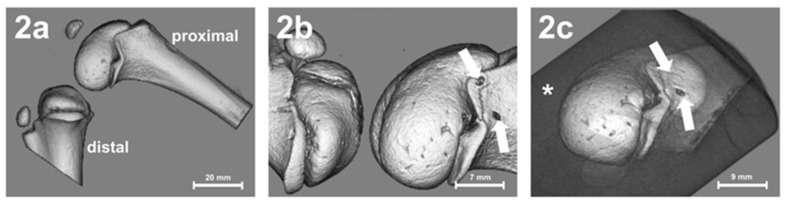
Micro-CT imaging of the left knee joint: (**a**) before ablation, (**b**) after ablation, and (**c**) after embedding in synthetic resin (*). Two ablation lesions (marked by white arrows). Error bars with units in mm.

Micro-CT diagnostics were performed to determine the localization of the growth plates, as well as the possible effect of thermal ablation. Micro-CT allows for differentiation between the cortical and trabecular bone. For this reason, micro-CT diagnostics were performed before and after thermal ablation in order to be able to visualize the effects image morphologically and after embedding in synthetic resin (Technovit^®^ 9100; Heraeus Kulzer GmbH; Wehrheim, Germany) (see Figure 1a1–a6 and Figure 2).

### 2.4. Extent of the Lesion as Measured by Volume Measurement Using Micro-CT

The extent of the lesions generated using thermal ablation was determined by volume determination of the lesions using micro-CT imaging. For micro-CT imaging, three micro-CT images (in vivo micro-CT systems Quantum FX (Perkin Elmer LAS GmbH; Rodgau, Germany)) were taken on each lamb bone before and after ablation, as well as after embedding in synthetic resin (Technovit^®^ 9100; Heraeus Kulzer GmbH; Wehrheim, Germany). To acquire the images, the piece of bone was placed in the specimen holder, fixed, and positioned centrally in the scan shaft. The resulting volume data sets were analyzed using the 3D rendering and analysis software Scry version 7.0, a fast and routine reporting system of Scry Analytics (Saratoga, CA, USA).

The quantitative evaluation of the visible lesions was performed using the measuring instruments of the program. The tissue changed with the electrode tip, which corresponded in shape to an ellipse-shaped cylinder. This finding was used to measure the volume of visible change. By determining the coordinates (*x*, *y* and *z*) of a point at the base and tip of the cylinder, the height (*h*) could be calculated as follows:

$$h=\surd ({\left(x1-x2\right)}^{2}+{\left(y1-y2\right)}^{2}+{\left(z1-z2\right)}^{2})\times \mathrm{pixel}\;\mathrm{size}$$

The pixel size here was 0.08 (mm/pixel). To calculate the elliptical basic shape, the length and width of the elliptical surface were measured at three points each and the average value was calculated. The volume (*V*) could then be represented by the following formula:

$$V=h\times \pi \times \left(\frac{\emptyset \mathrm{length}}{2}\right)\times \left(\frac{\emptyset \mathrm{width}}{2}\right)$$

Furthermore, the minimum and maximum distance from the center of the visible lesion to the growth plate were measured. The average (∅) of the values obtained from two measurements was taken. The length and width were determined similar to the height (see above).

### 2.5. Histology

**Figure 3:**
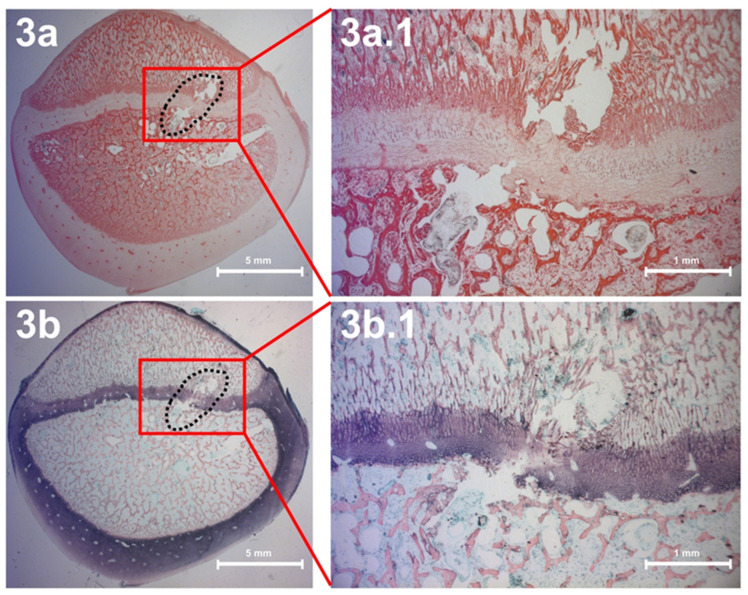
Sagittal section showing thermal lesions after electrocautery on the left side of the proximal tibia using H&E staining (**a**) and van Gieson staining (**b**). (**a**) The black dotted line shows thermal ablation lesion channel. The red box shows the magnification of the lesion channel ((**a**): scale bar 5 mm; (**a.1**): enlargement, scale bar 1 mm). (**b**) The same sectioning in van Gieson staining. The black dotted line shows the lesion channel. The red box shows the magnification of the lesion channel ((**b**): scale bar 5 mm; (**b.1**): enlargement, scale bar 1 mm).

In preparation for the subsequent histological processing (see Figure 3 below), all soft tissues were removed from the bones of the specimens and the joints were disarticulated. The samples were then dehydrated for about 2 months in an ascending ethanol series starting with 4% formalin fixation (ChemSolute^®^; Th. Geyer Ingredients GmbH & Co. KG; Höxter, Germany), and subsequently fixed in a methyl methacrylate embedding system (Technovit^®^ 9100; Heraeus Kulzer GmbH; Wehrheim, Germany). Coronal and sagittal sections (39.84 µm) were taken using an Optical Coherence Tomography (OCT)-image Guided Laser Microtome (LLS ROWIAK LaserLabSolutions GmbH; Hannover, Germany) until three sections were obtained containing the growth plate and a control incision approximately 1 cm offset from the lesion. The slices were stained with hematoxylin and eosin (H&E) as well as van Gieson (staining protocol see below). The staining controls of the histological sections were performed microscopically (Leica MZ75, Leica Mikrosystems GmbH, Wetzlar, Germany).

Staining was started by hydration of the sections for 2 × 30 min in a mixture (1:1) of methyl metacrylate (Merck KGaA; Darmstadt, Germany) and xylene (Carl Roth GmbH and Co. KG; Karlsruhe, Germany), followed by 10 min incubation in 99% Ethanol (Carl Roth GmbH and Co. KG; Karlsruhe, Germany), 5 min in 75% ethanol (Carl Roth GmbH and Co. KG; Karlsruhe, Germany), and finally for 5 min in demineralized water. Staining took place for 60 min in Mayer’s Hematoxylin solution (Carl Roth GmbH and Co. KG; Karlsruhe, Germany), followed by destaining (10 min) in running water (to remove unspecific background staining) and by subsequent incubation (10 min) in an Eosin−Phloxine solution (1 g/L Eosin and 0.2 g/L Phloxine in 2-Propanol (Morphisto GmbH; Offenbach on the Main, Germany). Dehydration of the slides was done for 30 s in 75% ethanol (Carl Roth GmbH and Co. KG; Karlsruhe, Germany), followed by 2 min of 99% ethanol (Carl Roth GmbH and Co. KG; Karlsruhe, Germany), and was finished in 1 min of xylene (Carl Roth GmbH and Co. KG; Karlsruhe, Germany). The covering of the slides was done with Histokit II (Carl Roth GmbH and Co. KG; Karlsruhe, Germany).

The staining protocol of the van Gieson staining was started by heating it up in Sandersons Rapid (Bone) Stain (SRS) (Dorn&Hart Microedge Inc.; Loxley, AL, USA), followed by putting a drop of SRS on a plastic section covering the complete section and leaving it to act it for 20 min. This was followed by washing the SRS with distilled water and drying with tissue paper for 20 s and covering the section with a drop of van Gieson Solution (Carl Roth GmbH & Co. KG; Karlsruhe, Germany) for 45 s. Finally, the slides were washed with distilled water and dried with tissue paper for 20 s, before the slides were then finally dipped into xylene and coverslipped with Histokit II (Carl Roth GmbH and Co. KG; Karlsruhe, Germany).

### 2.6. Statistics and Graphics

Statistical analysis was not required in the main part of the study, as no conditions were compared in our study.

Measurements were taken three times by two persons for each location, independently. The mean values of these six measurements are presented in Table 1. The graphics and variance calculation were performed with the statistics software GraphPad Prism 9 (version 9.1.0 for mac; GraphPad Software, San Diego, CA, USA).

## 3. Results

To investigate the precision of electrocautery at the growth plate, we positioned thermal ablation under radiographic control at the growth plates of a stillborn linseed lamb and afterwards it was scored by micro-CT and histology to discover whether the targeting of the lesion was correct.

### 3.1. Accuracy of the Electrocautery Treatment

Thermal ablation aimed at the growth plate was carried out under standardized radiographic examination in two planes, and was subsequently controlled using micro-CT. As revealed by micro-CT imaging, in 12 out of 16 cases (75%), the growth plates were properly hit by the electrocautery tips, whereas in the other four cases, including the distal femur, the distal tibia, the proximal tibia, and the distal femur, the growth plates were missed by 4.69 mm, 0.89 mm, 1.78 mm and 1.97 mm, respectively. The average distance from the center of the lesions with respect to the position of the growth plates was 1.12 ± 1.07 mm, as measured by micro-CT. The results are displayed in Table 1.

### 3.2. Spatial Effect of the Thermal Ablations as Determined by Histology and by Micro-CT

In order to visualize the effects of thermal ablation hits on the growth plates after thermal ablation, we sectioned the corresponding regions of the bone and examined them microscopically after hematoxylin and eosin and van Gieson staining (see Material and Methods). Figure 3 shows the effect of targeted thermal ablation on the growth plate region of the proximal tibia. This indicates that there was indeed disrupted tissue in the area of the growth plate that was considered by electrocautery. In addition, Figure 3 documents that the lesion was confined to a narrow area and was not significantly larger than the region to which the heat was applied.

**Figure 4:**
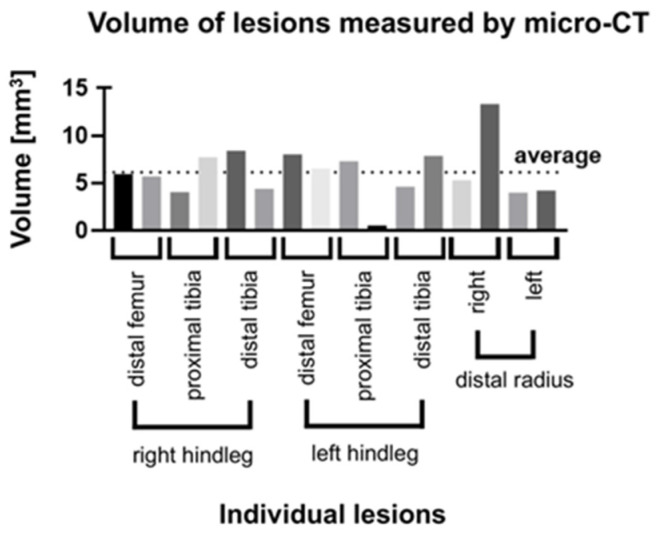
Volume of lesions measured using micro-CT. The volume of lesions are shown [mm^3^] on the *y*-axis and the lesion locations on the different extremities are shown on the *x*-axis. The black dashed line indicates the average.

To determine the extent of the thermal lesions, i.e., the three-dimensional effect to which we refer to as “unit of volume“, we used micro-CT imaging. The results show that the lesion volumes (Table 1) were variable and the mean volume determined using micro-CT was 6.14 ± 2.82 mm (Figure 4). The calculated variance of the volumes was 7.85 (mm^3^)^2^.

## 4. Discussion

In this study, the extremities of a stillborn lamb were used as a preclinical model for the effects of thermal ablation on growth plates using electrocautery. For thermal ablation to have an optimal therapeutic effect on epiphyseal growth, it is necessary to have a high hit rate in the relevant region, as well as to inactivate a sufficiently large area within the growth plate in order to achieve the required growth arrest.

The presented thermal ablation approach has the potential to replace or to be used in addition to conventional surgical methods [4,5], with possibly fewer side effects due to incision-related scar tissue, hematoma, and swelling, as well as implant displacement and secondary surgery. Because of the local effect of thermal ablation, as demonstrated in the histological examination, a controlled hemiepiphyseodesis with subsequent axial correction seems very possible. For epiphysiodesis, it is important to ultimately destroy enough of the epiphysis region. However, there is still no standard protocol of how much needs to be deleted from the growth plate in order to achieve the intended effect. Nevertheless, the described approach provides the opportunity to more accurately target and safely destroy the region of the epiphysis that needs to be disrupted.

During the intervention, standardized anterior−posterior and lateral radiographic views were performed to ensure optimal placement. However, micro-CT imaging revealed only 75% of the optimal placement using the gold standard procedure. This may be due to the irregular shape of the growth plate and the penetration depth of the electrocautery needle. The localization and the extent of the generated lesions from the electrocautery were analyzed via the lesion volume, and were also determined by micro-CT imaging. Notably, however, this technique cannot routinely be used in children due to adverse effects with increased radiation exposure.

The lesions after heat treatment were, on average, about four-fold larger compared with the treatment without the heat applied (see Table 1). Thus, the lesions were caused by a combination of physical forces and heat, which resulted in deeper penetration. Importantly, however, thermal ablation caused only the disruption of a small area as revealed by histology, i.e., the procedure described here is indeed suitable for disrupting even small areas within the growth plate. The different distances of the inserted tip of the electrocautery tool to the growth plate are also listed in Table 1, showing that within a distance of about 5 mm between the inserted tip of the electrocautery tool and the growth plate, a lesion in the growth plate has been caused. Thus, heat not only acts at the insertion site but also affects the surrounding area.

A reason for the observed differences of the volume of the individual lesions (calculated variance 7.85 (mm^3^)^2^) is likely due to the fact that growth plate and cartilage tissue are difficult to distinguish with this imaging modality. It should also be noted that in one of the two measurements of thermal ablation in the distal radius region, the treatment appeared to be more effective than observed in all other experiments (Figure 4). We suspect that this difference is due to the experimenter and to a one-time event, since all other values show a comparably small difference between the two experiments at the same growth plate. However, this finding also shows that the surgical approach with thermal ablation depends on the skill of the experimenter. Notably, however, the overall lesion volumes were remarkable similar to the mean value of all measurements, i.e., 6.14 ± 2.82 mm^3^, even when the two outliers in position PTPL L2 and DRAR L2 were included in the dataset (see Table 1 and Figure 4).

Previous studies in rabbits and pigs have shown that radio-frequency ablation affects bone growth differentiation [7], i.e., growth inhibition was achieved by radio-frequency coagulation of the physis [8]. These results supported earlier results on rabbits, suggesting that radio-frequency ablation may indeed be useful for epiphysiodesis [9,10]. More recently, Shiguetomi-Medina et al. [11] performed epiphysiodesis using radio-frequency ablation in vivo at a high temperature due to high-frequency radio waves [12]. They showed that thermal epiphysiodesis using radio-frequency coagulation disrupts the physeal morphology and causes the formation of bone bridges at the ablation sites [11]. However, damaged tissue next to the newly formed bone bridges was characterized by disorganization and fibrosis [11]. Complementary therapeutic approaches have been used to stimulate rabbit growth plates by high-energy extracorporeal shock waves, which stimulated the growth of the rabbit epiphysis [13]. Furthermore, high-dose ultrasound had a similar effect [14]. However, it had profound pathological effects on the growing bone, whereas a reduction in therapeutic ultrasound doses had no effects on the growing bone [14].

The potential of the thermal ablation technique presented here for epiphysiodesis and hemi-epiphysiodesis with respect to clinical implications cannot be assessed. Thermal ablation has the advantage over standard percutaneous techniques in that it can be performed in a minimally-invasive manner and no follow-up procedures, such as a subsequent metal removal, are required. However, as clinical data are not yet available for the proposed thermal ablation therapy, its potential and disadvantages compared with the current treatments cannot be properly assessed. These would have to be investigated in more detail in further studies, including preclinical in vivo studies, with the newly introduced lamb model organism. It also needs to be examined whether thermal ablation needs to be performed at different growth plate sites in order to be effective in bone growth modulation or whether it is sufficient to perform the treatment at one insertion site, including the use of larger-sized electrocautery tips. These aspects need to be investigated in order to see whether thermal ablation is a superior treatment to, for example, percutaneous drilling, which is also a minimally invasive technique.

The obvious limitation of our study is that we did not examine the effects of the applied thermal ablation in a living sheep organism. This application is necessary in order to assess the consequences concerning the outcome of thermal ablation treatment on directed bone growth. This limitation of our pilot study will be overcome by future studies designed to examine the consequences of thermal heat to the epiphyseal growth plate, i.e., in order to see how bone growth can be modulated.

Importantly, however, our results establish that thermal ablation has the potential to be used as a minimally invasive alternative or in addition to percutaneous drilling, metal implantation, and the even more extensive surgical epiphyseodesis methods.

## 5. Conclusions

Our pilot study suggests that thermal ablation may be used as a minimally invasive approach to destroy distinct areas of the epiphyseal growth plate. Using standardized radiographic procedures, perfect localization was achieved in 75% of cases and tissue disruption only occurred locally. However, preclinical in vivo studies are necessary in order to determine the effects in a growing organism.

## Figures and Tables

**Table 1:** Results of targeting, distance measurements, and volume determination.

	Micro-CT		
**Localization**	**Distance of** **Electrode Tip** **to** **Growth Plate** **[mm]**	**Volume of the Lesion [mm^3^]**	**Mean Value L1/L2** **[mm^3^]**
DFPR L1	4.69	5.97	5.58
DFPR L2	0.78	5.73	
PTPR L1 PTPR L2 DTPR L1	0.60 0.89 1.78	4.07 7.73 8.43	5.90 6.43
DTPR L2	1.32	4.42	
DFPL L1	1.97	8.05	7.31
DFPL L2	0.68	6.56	
PTPL L1 [PTPL L2] ^##^	0.97 [0.00] ^##^	7.32 [0.55] ^##^	3.94 ^#^
DTPL L1 DTPL L2	0.78 0.87	4.64 7.88	6.26
DRAR L1 DRAR L2	0.45 1.19	5.31 13.35	9.33
DRAL L1	0.46	4.01	4.13
DRAL L2	0.48	4.24	
**Average ± SD**	**1.12 ± 1.07**	**6.14 ± 2.82**	**6.14 ±** **1.72**
**CONTROLS**			
DFPR L1 DFPR L2 DTPR L1 only one lesion DFPL L1 only one lesion	4.29 4.36 0.24 - 0.69 -	0.63 0.50 1.54 - 2.56 -	0.57 - 1.54 - 2.56 -
**Average ± SD**	**2.39 ± 1.94**	**1.31 ± 0.82**	**1.58 ± 0.79**

DFPR = distal femur posterior right; PTPR = proximal tibia posterior right; DTPR = distal tibia posterior right; DFPL = distal femur posterior left; PTPL = proximal tibia posterior left; DTPL = distal tibia posterior left; DRAR = distal radius anterior right; DRAL = distal radius anterior left; L1 = lesion 1; L2 = lesion 2. ^#^ mean value was determined although the second value is likely due to an experimental error. [] ^##^ as the difference between heating and only insertion of the electrocautery tip is close to four-fold, we assume that this measurement is due to an experimental error, i.e., insertion of the tip only, without heat applied.

## Data Availability

The data that support the findings of this study are available upon request from the corresponding author.
